# Comparison of entomological impacts of two methods of intervention designed to control Anopheles gambiae s.l. via swarm killing in Western Burkina Faso

**DOI:** 10.1038/s41598-022-16649-7

**Published:** 2022-07-20

**Authors:** Simon P. Sawadogo, Abdoulaye Niang, Sean L. Wu, Azize A. Millogo, Jane Bonds, Mark Latham, Roch K. Dabiré, Allison Tatarsky, Frederic Tripet, Abdoulaye Diabaté

**Affiliations:** 1 Institut de Recherche en Sciences de La Santé, Bobo-Dioulasso, Burkina Faso; 2 Division of Epidemiology and Biostatistics, School of Public Health, University of California, Berkeley, CA 94720 USA; 3 Institut de Sciences Des Sociétés (INSS), Ouagadougou, Burkina Faso; 4 Bonds Consulting Group LLC, Florida, USA; 5 Manatee County Mosquito Control, Florida, USA; 6 Malaria Elimination Initiative, University of California, San Francisco, CA USA; 7 Centre for Applied Entomology and Parasitology, School of Life Sciences, Keele University, Staffordshire, UK

**Keywords:** Biological techniques, Ecology, Medical research

## Abstract

Outdoor biting constitutes a major limitation of current vector control based primarily on long-lasting insecticidal nets and indoor residual spraying, both of which are indoor interventions. Consequently, malaria elimination will not be achieved unless additional tools are found to deal with the residual malaria transmission and the associated vector dynamics. In this study we tested a new vector control approach for rapidly crashing mosquito populations and disrupting malaria transmission in Africa. This method targets the previously neglected swarming and outdoor nocturnal behaviors of both male and female *Anopheles* mosquitoes. It involved accurate identification and targeted spraying of mosquito swarms to suppress adult malaria vector populations and their vectorial capacities. The impact of targeted spraying was compared to broadcast spraying and evaluated simultaneously. The effects of the two interventions were very similar, no significant differences between targeted spraying and broadcast spraying were found for effects on density, insemination or parity rate. However, targeted spraying was found to be significantly more effective than broadcast spraying at reducing the number of bites per person. As expected, each intervention had a highly significant impact upon all parameters measured, but the targeted swarm spraying required less insecticide.

## Introduction

Despite several initiatives undertaken to reduce its related burden in the last two decades^1^ malaria remains one of the most important public health problems especially in many sub-Saharan African countries^1^. Due to the global control programme, malaria prevalence and mortality have drastically decreased from 2000 to 2015 before being almost stable until 2019 with a trend of increase in 2020 and 2021. The slight increase in malaria cases has been attributed to the slackening of interventions during the COVID-19 pandemic period. Although several factors might have contributed to the 15 years sharp decline, vector control interventions—mainly Indoor Residual Spraying (IRS) and scale-up of Long-lasting Insecticide-treated Nets (LLINs), made the greatest contributions^2–4^. LLINs and IRS are known to be effective killers of endophilic mosquito species. However, several studies demonstrated that residual malaria transmission might be maintained by bites which occur when people are not protected by these conventional tools^5–7^. Some vector species have adapted to seeking hosts outdoors, thereby escaping the LLINs or IRS before absorbing a lethal dose of insecticide. For example, *Anopheles arabiensis*, which now predominates most residual transmission systems across Africa, has been observed to be far less responsive to indoor interventions than its sibling species *An. gambiae* and *An. coluzzii*, because, even when indoors seeking a human blood meal, they tend to exit earlier than usual, especially when the people indoors use bed nets^8^. The increasing importance of outdoor biting constitutes a major limitation to current vector control tools based primarily on LLINs and IRS, both of which are indoor interventions^9^. Consequently, malaria elimination will not be achieved unless additional tools are found to deal with the residual malaria transmission and the associated vector dynamics. The malaria eradication research agenda initiative (malERA), in its 2011 reports, defined the major research targets that would be needed to sustain and improve effectiveness of currently available control tools^10^. Emphasis was placed on developing interventions that affect mosquito behaviours not effectively targeted by LLINs and IRS^9^. Apart from endophagous and endophilic behaviour, there are many other activities of mosquitoes occurring mainly outdoors which escape the indoor interventions. These activities include the search for oviposition sites, the search for nectar sources, outdoor resting and swarming^11–13^. Therefore, new approaches might focus on other aspects of mosquito biology and ecology or nocturnal activities such as outdoor-biting and mating^14,15^.

*An. gambiae* s.l. mosquitoes, as many other malaria vectors, mate in flight at sunset. Males gather over specific landmarks known as swarm markers to attract conspicuous females. It is not known why males are attracted to these landmarks, but visual cues seem to play an important role in selecting the swarming sites^16^. Generally, these landmarks create either a dark/light contrast on the ground or they interrupt the regularity of a smooth landscape. Swarms could be induced to appear in places where they were previously absent or repelled away from traditional markers by means of artificial markers. Interestingly, males systematically use the same sites to swarm over time and even after several years^17^. This characteristic behaviour to fly toward a landmark for mating is found across malaria vector species and across geographic regions in Africa including Burkina Faso, Mali, Benin^17–20^, Sudan, Cameroon, the Gambia, Sao Tome et Principe, Mozambique and Tanzania^21–25^. The concentrations of males, predictability and accessibility of the swarming sites, and the fact that swarms can be artificially manipulated, make them an easy control target. Moreover, the tight clustering of the target provides opportunities for controlling the vectors with reduced dose space-spray applications over the traditional broadcast space spray. Indeed, in a preliminary experiment designed to assess the potential impact of swarm-killing on *An. gambiae* s.l. populations in an intervention village versus a control in the Vallee du Kou, mass killing of swarming mosquitoes resulted in a ~ 80% decrease female mosquito indoor densities^26^. A significant impact on the age structure of male population was observed, and the proportion of younger males increased from 20 to 70% indicating that the intervention could preferentially eliminate old males.

For the past twenty years, ground-breaking studies on mosquito mating behaviour conducted mostly in west Africa have provided fresh insights into the mechanisms of assortative mating between sibling species of *An. gambiae* complex^27^. Typically, these studies were mostly motivated by the need for advancing novel tools for genetic control of malaria mosquitoes^16,28^, In contrast here, we proposed a different approach, which will attempt to suppress mosquito populations by identifying and directly targeting swarms with highly effective insecticides, using proven spraying techniques delivered by trained volunteers recruited from the beneficiary communities^16,26^. Thus this study targeted the previously neglected swarming and outdoor nocturnal behaviors of both male and female *Anopheles* mosquitoes. It first required accurate identification and targeted spraying of mosquito swarms to suppress adult malaria vector populations and their vectorial capacities. A key objective in the evaluation of this intervention was to understand its impact on mosquito densities and secondary entomological outcomes such as reduced survival, change in age structure of females, and a decrease in entomological inoculation rate (EIR). Another objective, was to assess whether these outcomes could be achieved through the involvement of trained community volunteers. The impact of targeted spraying was compared to that of broadcast spraying, with both methods evaluated simultaneously.

## Materials and methods

### Study sites and swarm characterization

**Figure 1:**
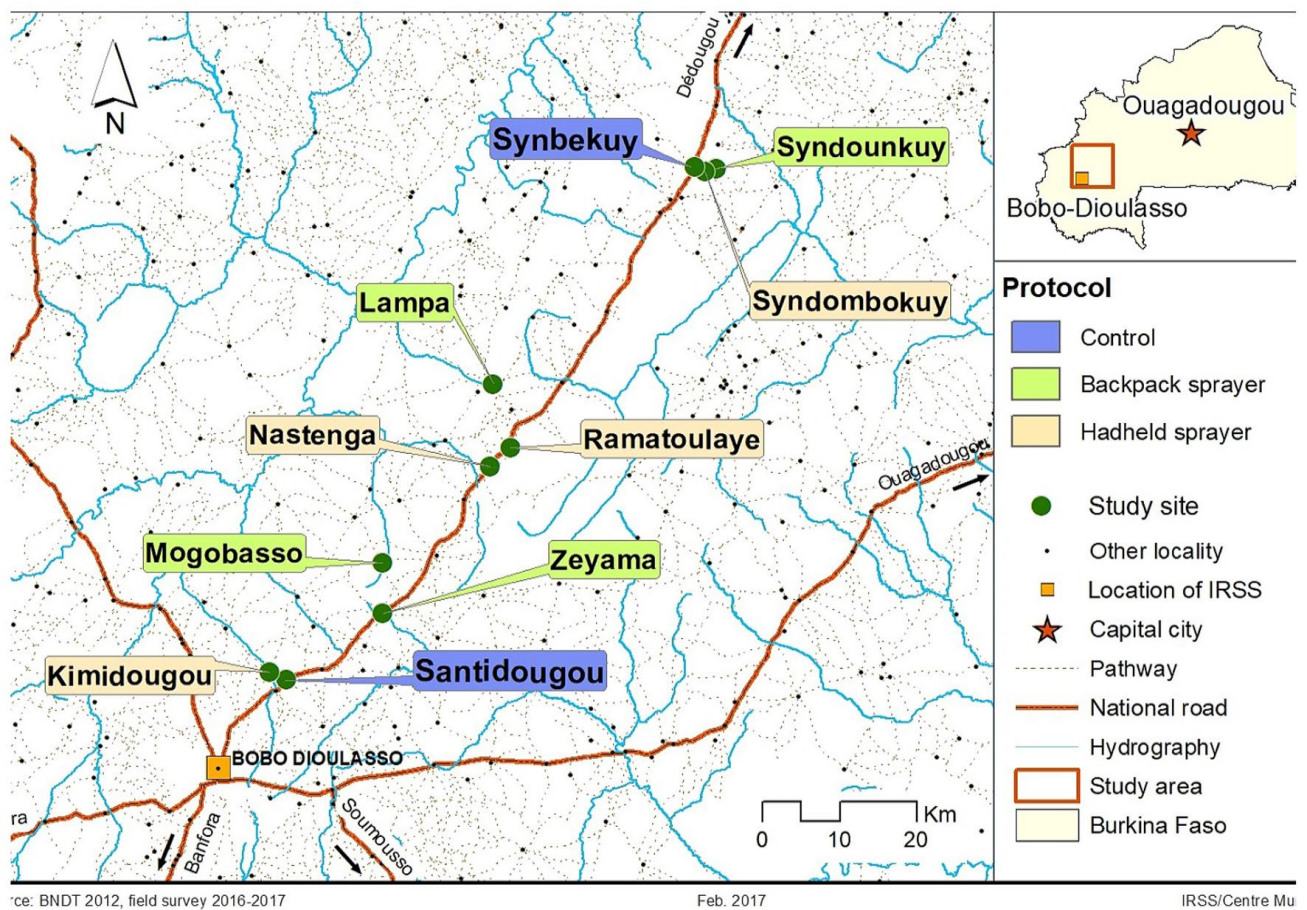
Localization of the study sites in south-western Burkina Faso. This map was created under QGIS version 2.18 Las Palmas. link: https://changelog.qgis.org/en/qgis/version/2.18.0/

The survey was conducted in 10 villages in south-western Burkina Faso especially around the district of Bobo-Dioulasso, Santitougou (N11° 17′ 16″, W4° 13′ 04″), Kimidougou (N11° 17′ 53″; W4° 14′ 11″), Nastenga (N10.96871; W003.23477), Zeyama (N10.87638; W 003.26145), Mogobasso (N11° 25′ 31″, W4° 06′ 08″), Synbekuy (N11° 53′ 28″, W3° 44′ 02″), Ramatoulaye (N11° 33′ 39″, W3° 57′ 05″) Syndombokuy (N11° 53′ 06″, W3° 43′ 19″), Lampa (N11.16464; W 003.6374) et Syndounkuy (N11.14541; W 003.05141) (Fig. 1). All villages are located north of Bobo-Dioulasso, on the national road 10 (N10), ranged from 20 and 90 km. The region is characterised by wooded savannah located in south-western Burkina Faso, and the mean annual rainfall is about 1200 mm. The rainy season extends from May to October and the dry season from November to April. Malaria transmission in the area extends from June to November. However, residual transmission may occur beyond this period in specific locations. *An. gambiae* is the major malaria vector following by *An. coluzzii* and *An. Arabiensis*. Villages were chosen to represent similar ecological and entomological settings, they are middle sized and relatively isolated from one another.

Spray Application Against Mosquito Swarms (SAMS) consisted of spraying diluted insecticide (Actellic 50: tap water with 1:20 concentration) at dusk by trained volunteer teams. They used the innovative technology of targeted swarm spraying with handheld sprayers and conventional broadcast space spray with backpack sprayers to achieve maximum effect. The spraying activities were conducted in eight of the ten villages. The target swarm spray was used in the four villages Kimidougou, Nastenga, Ramatoulaye and Syndombokuy. The broadcast space spray was applied in four other villages, Zeyama, Mogobasso, Lampa and Syndounkuy. The two remaining villages, Santidougou and Synbekuy were chosen as controls (Fig. 1). In each village, the potential swarm markers and the positive swarm sites were identified and geo-referenced using GPS. All concessions also were geo-referenced and labelled using paint.

### Procedure of the intervention

#### Targeted swam spraying using handheld sprayers

**Figure 2:**
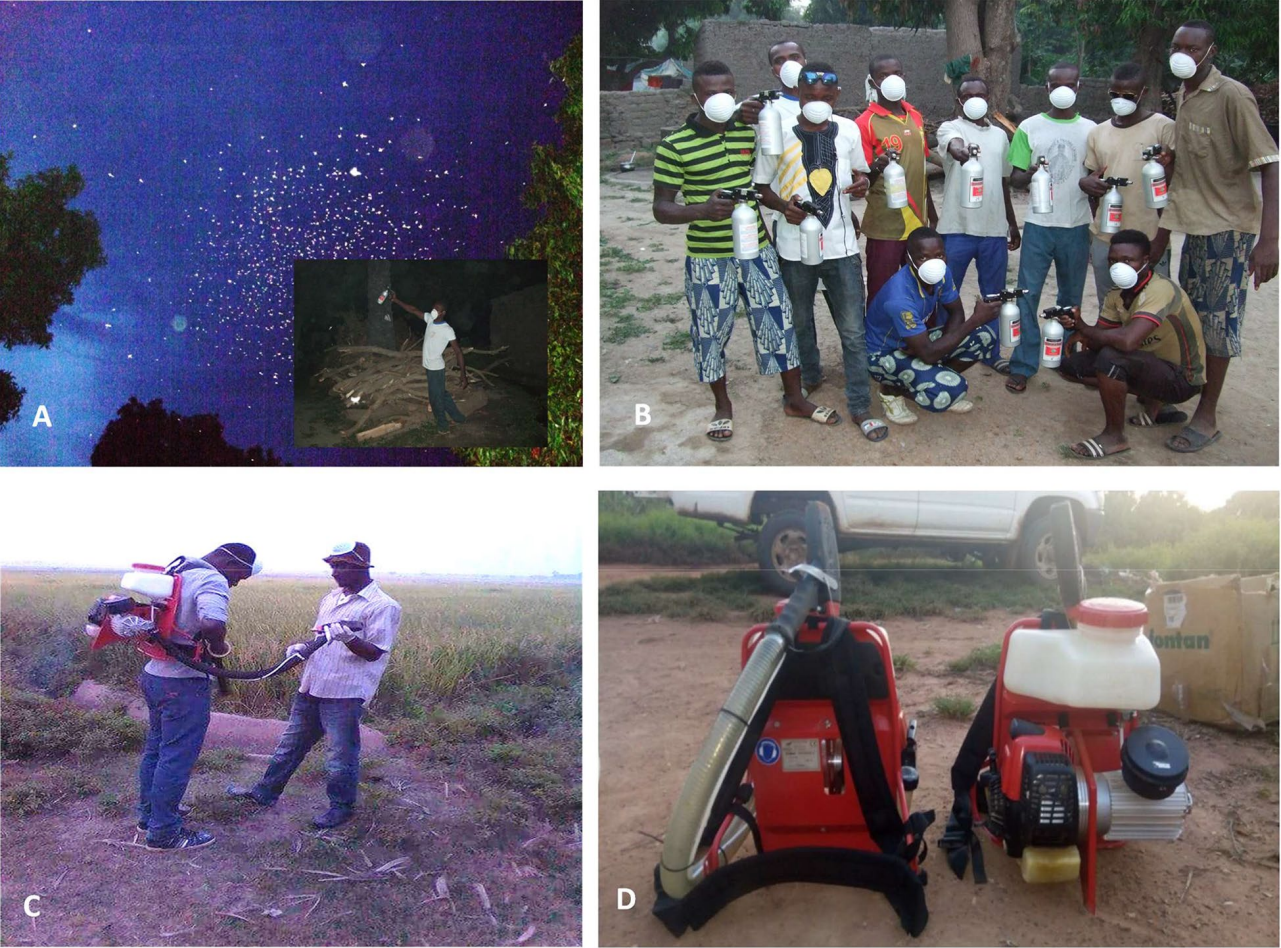
Volunteer spraying swarms using handheld sprayers (**A**,**B**). Backpack spraying activities (**C**,**D**).

Targeted swarm spraying was carried out in four villages. Members of each team and volunteers from the selected villages were trained to target the swarms and apply an appropriate amount of spray each time. After the pre-intervention phase, all swarm sites scattered through the villages were repaired and swarm characteristics recorded. At 30 min before dusk (the estimated swarming time), a volunteer was placed in each compound with a sprayer. The objective of each volunteer was to destroy any swarm in the compound by applying insecticide with the handheld sprayer (Fig. 2A,B). Screening of the compound was continued for about 30 min until it was dark and no mosquitoes were visible. A single operator was able to effectively target 5 to 10 swarms per spray evening, depending on the distribution of swarms across the village. Spraying was carried out for 10 successive days throughout each village. The period of spraying approximately covered the period of pre-imaginal mosquito stages and was renewed after 45 days. The quantity of insecticide used was measured daily, in order to determine with precision the total quantity of insecticide used during targeted spraying.

#### Conventional broadcast spraying using Backpack sprayers

The broadcast spraying was also carried out in 4 villages but, unlike the targeted spraying, there was no direct targeting of swarms. At swarming time (estimated around 30 min at dusk) two volunteers with backpack sprayers ran through the entire village along paths between the compounds while spraying insecticide (Fig. 2C,D). As with the targeted spraying procedure, the broadcast spraying was carried out for 10 successive days in all 4 villages simultaneously, and spraying recommenced after 45 days. The quantity of insecticide used was measured daily, in order to determine with precision the total quantity of insecticide used during targeted spraying.

#### Evaluation of the intervention

A year prior to the intervention, baseline entomological data was collected in both villages to estimate mosquito density, human biting rate, female insemination rate, age structure of females and entomological inoculation rate^29^. The same parameters were evaluated immediately before and after intervention. The pre- and post-intervention evaluation of the abovementioned parameters were carried in both control and intervention villages at the same time. In both pre-intervention and post-intervention phases, two methods of mosquito collection were performed in each village, the human landing catch (HLC), indoor and outdoor in 4 houses for 4 successive nights, the pyrethroid spray catch (PSC) in the same10 houses and 10 randomly selected houses. To identify these, all houses in each village were coded and these codes were used to randomly select those to be sampled. All sampled sites were mapped using a global positioning system (GPS). Collected anopheline mosquitoes were sorted by taxonomic status, physiological status, and sex. Approximately, the ovaries of 200 females/month/village (100 females indoor and 100 females outdoor) were dissected to determine the physiological age, and parous females were subsequently subjected to ELISA assays to determine *Plasmodium* sporozoite rates. Data produced from indoor and outdoor mosquito collections were then used to estimate mosquito densities, their spatial distribution, produce a map identifying hotspots where the highest mosquito densities and biting occurred within the village, female age structure and quantify the intensity of malaria transmission. The impact of the spray was measured to see how it affected each of these parameters in the intervention villages compared to the controls.

### Statistical analysis

The resting mosquito abundance was assessed as the number of mosquitoes per house, the human biting rate assessed as the number of bites per person per night, the parity rate assessed as the percentage of parous females, and the insemination rate assessed as the percentage of the inseminated females. The list above defined the key entomological parameters to determine the dynamic of *An. gambie* s.l. populations and malaria transmission. The generalized estimating equation (GEE) method was used to estimate population averaged effect of intervention on various outcome measurements. As the GEE models do not require distributional assumptions but only specification of the mean and variance structure, they are more robust against misspecification of higher-order features of the data, and are useful when the main interest is in population averaged effects of an intervention or treatment. However, because they do not use a full likelihood model, they cannot be used for individual-specific inference^30,31^. Despite this shortcoming, their robustness to different types of correlation structures in the data (due to temporal ordering of measurements, or other hierarchical structure in data) makes them attractive for analyses of this type. GEE models were run in **R** version 3.6.2^32^, using the package “geepack”^33^ for three datasets on insemination and parity rate, number of bites per person per night (NBPN), and density of adult male and female mosquitoes. To clean and plot the data the “tidyverse” family of **R** packages^34^ were used.

### Ethical considerations

This study did not involve human patients. The full protocol of the study was submitted to the Institutional Ethics Committee of the “Institut de Recherche en Sciences de la Sante” for review and approval (A17-2016/CEIRES). In accordance with the approval, presentations of the project were given to the study site villagers and requests for their participation were made. During these visits the objectives, protocol and expected results were explained and discussed, as well as the implications for the households willing to take part in this study. A written consent form was signed or marked with fingerprint by the head of the households before any activity could take place in his compound. Insecticides used in this study are approved for use by the Burkina Faso insecticide regulation authority.

## Results

### Impact of the intervention on swarms

**Figure 3:**
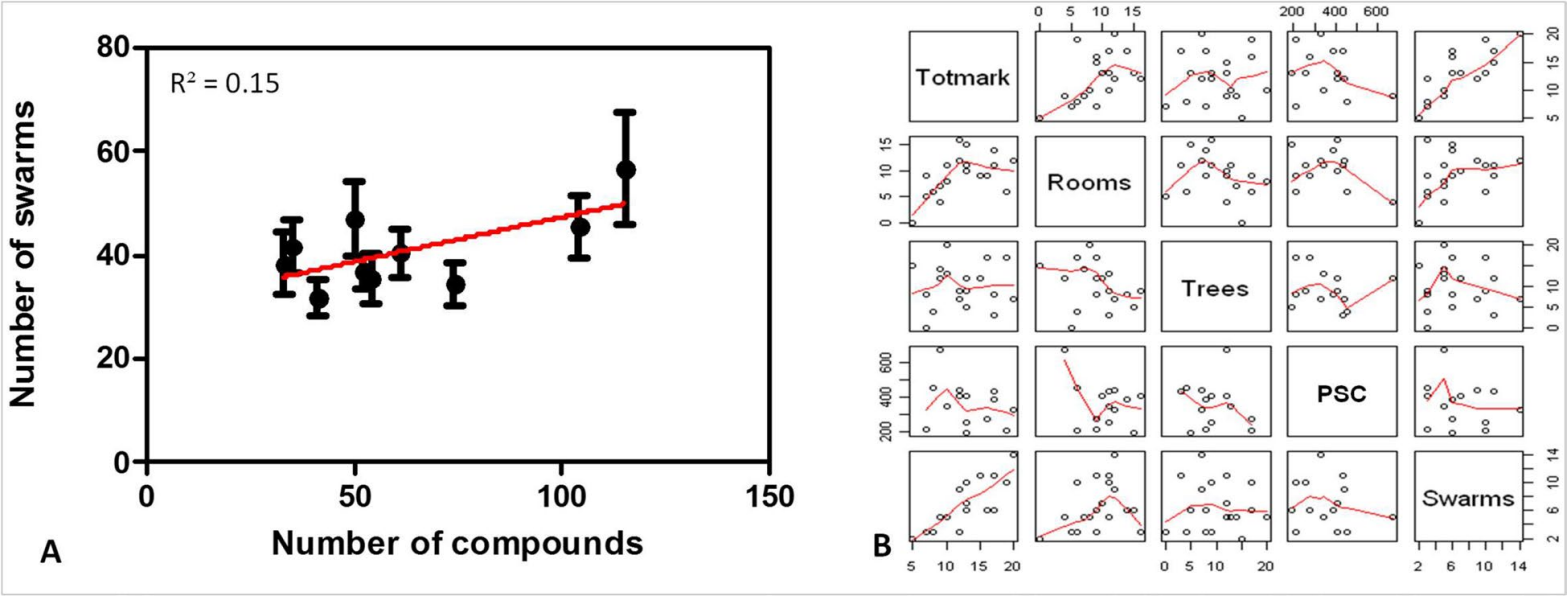
Correlation analysis between swarm abundance and the number of compounds (**A**) and the number of visual markers (**B**). *PSC* pyrethrum spray catch, *Totmark* total number of markers.

**Figure 4:**
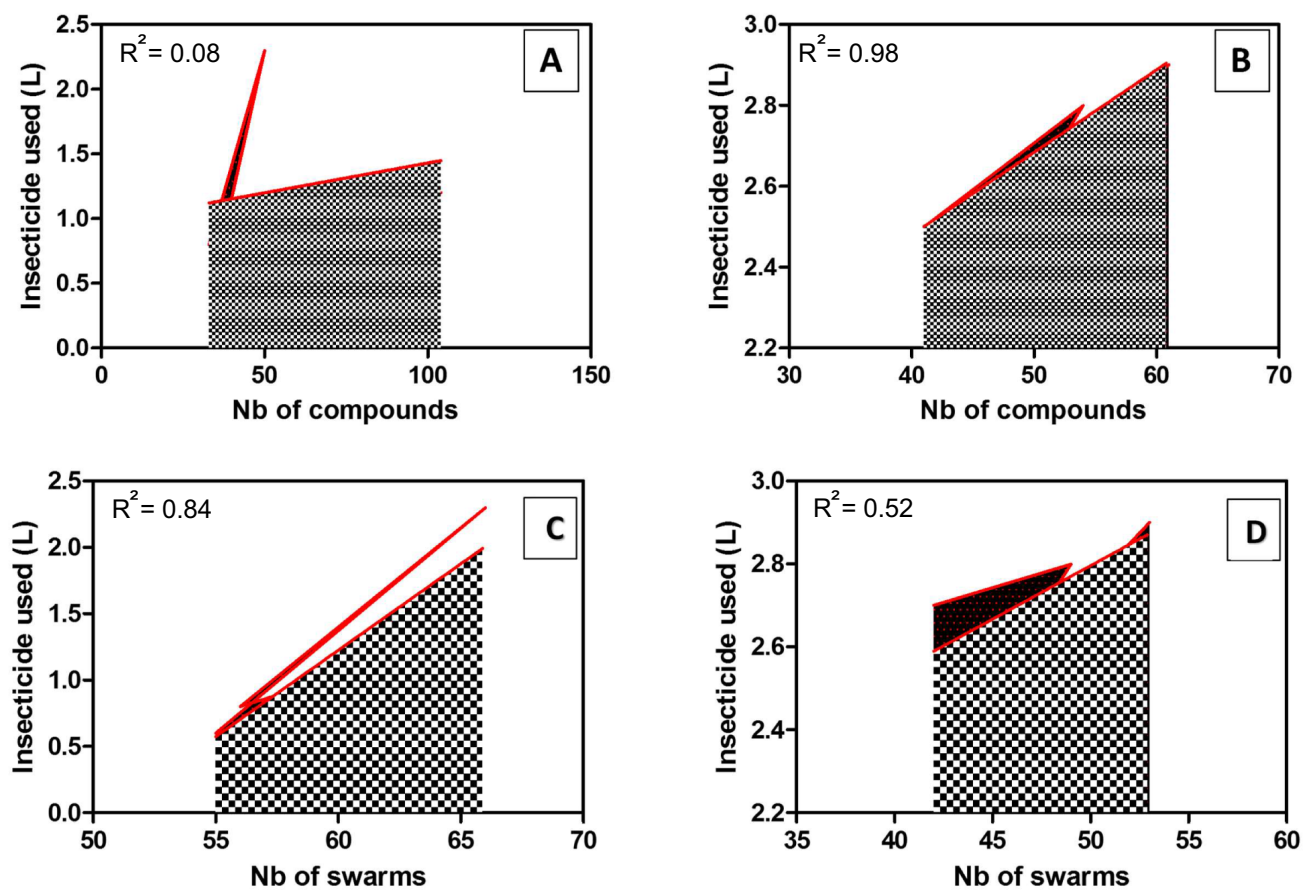
Relationship between number of compounds and the quantity of insecticide used in the targeted spraying (**A**) and Broadcast spraying (**B**). Relationship between the number of swarms and the quantity of insecticide used in the targeted spraying (**C**) and the Broadcast spraying (**D**).

The number of swarms was not correlated with the number of compounds (R^2^ = 0.15, P = 0.12, Fig. 3A). As demonstrated during the entomological baseline data collection in the year 1 (Niang et al. 2021), the number of swarms was correlated to the marker abundance (Fig. 3B), suggesting that the size of villages does not influence the quantity of insecticide used in targeted spraying. The correlation analysis performed between the number of compounds and the quantity of insecticide used showed statistically significant positive correlation (R^2^ > 0.98, P < 0.05) in the villages where broadcast spraying was used (Fig. 4B). However, no correlation was found in the villages where targeted spraying was used (R^2^ = 0.08, P = 0.80, Fig. 4A). Results suggest that with the broadcast spraying, the volume of insecticide increases with the size of the village. However, with the focalized space spray targeting swarms, the quantity of insecticide sprayed is not necessarily correlated to the size of the village, as small villages can generate more swarms. The quantity of insecticide used in targeted spraying appears to be density dependent, increasing with swarm abundance (Fig. 4C). However, the quantity of insecticide used in broadcast spraying was not significantly correlated to the number of swarms (Fig. 4D).

### Impact on the human biting rate, age structure of females and insemination rate

The data on the number of bites per person per night (NBPN) and insemination/parity rate were stratified by village, which was considered to constitute the clusters in data. As each village had multiple repeated measurements, an exchangeable working correlation structure to account for within-village correlation of observations was used, meaning that observations from the same village should be more similar than those between villages. It is stressed that the results are robust to misspecification of the correlation structure, although they may be less efficient. The generalized estimating equation (GEE) model considered the outcome to be the difference between measured NBPN pre- and post-intervention (using Gaussian model of mean structure) to consider possible differing effects of intervention type based on the pre-intervention measured NBPN, a main effect and interaction with intervention type for the mean-centered pre-intervention NBPN was included. Possible confounding by whether the measurement was taken indoors or outdoors, and by intervention round was also accounted for.

**Figure 5:**
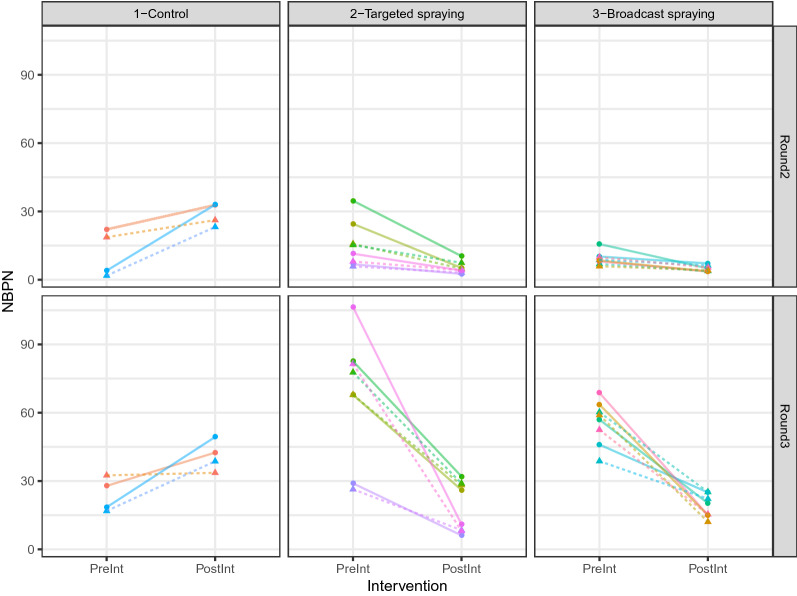
Plot of the raw NBPN data; stratified by intervention type (columns: control, targeted spraying, and broadcast spraying) and round (rows). Each village is assigned a unique color, and the change in NBPN between pre- and post-intervention measurements is plotted as a line. We found significant negative main effects of targeted spraying and broadcast spraying on NBPN (p-value = 0.037 and p-value = 0.026, respectively).

There were significant negative main effects of targeted spraying and broadcast spraying on NBPN (p-value = 0.037 and p-value = 0.026, respectively) when using a robust jackknife variance estimator to compute p-values and 95% confidence intervals (Fig. 5). No other terms in the GEE model had p-value < 0.05, although the term for intervention round had a p-value of 0.064. To check that GEE results were not anomalous, a standard GLM that did not account for within-village correlations was run. The GLM results were largely qualitatively similar, with similar values of regression coefficients but with extremely small p-values and overly narrow confidence intervals, restating the importance of accounting for within-village correlations.

**Figures 6:**
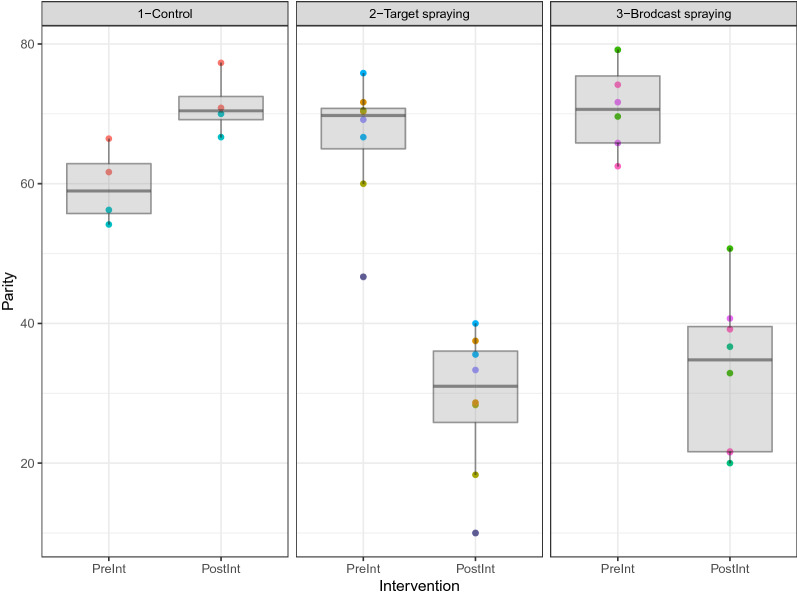
Plot of the parity rate. Each village is represented as a colored point and a boxplot is added to visually represent the dispersion of the data around its mean, shown pre- and post-intervention. We found highly significant effects from targeted and broadcast spraying in decreasing parity rate (p-value < 0.001).

**Figure 7:**
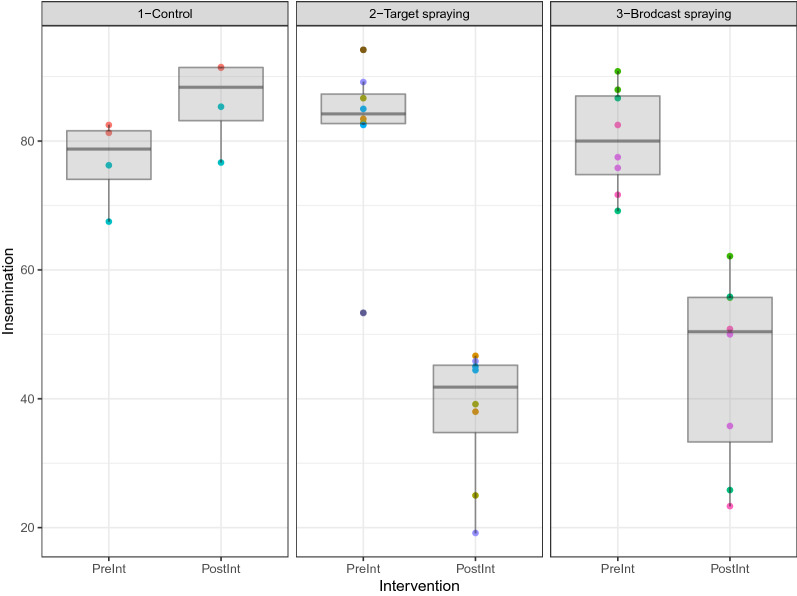
Plot of the insemination rate. Each village is represented as a colored point and a boxplot is added to visually represent the dispersion of the data around its mean, shown pre- and post-intervention. We found highly significant effects from targeted and broadcast spraying in decreasing insemination rate (p-value < 0.001).

A similar procedure was followed to analyze the insemination (Fig. 6) and parity (Fig. 7) rate data. Again, the GEE models considered the difference between pre- and post-intervention measurements as the outcome with a Gaussian mean structure and using robust jackknife variance estimator to compute p-values and 95% confidence intervals. Highly significant effects from targeted and broadcast spraying in decreasing insemination rate and parity rate (p-value < 0.001) were found. Results from GLM regression were very similar in both magnitude and estimated standard errors.

### Impact on the resting mosquito abundance

**Figure 8:**
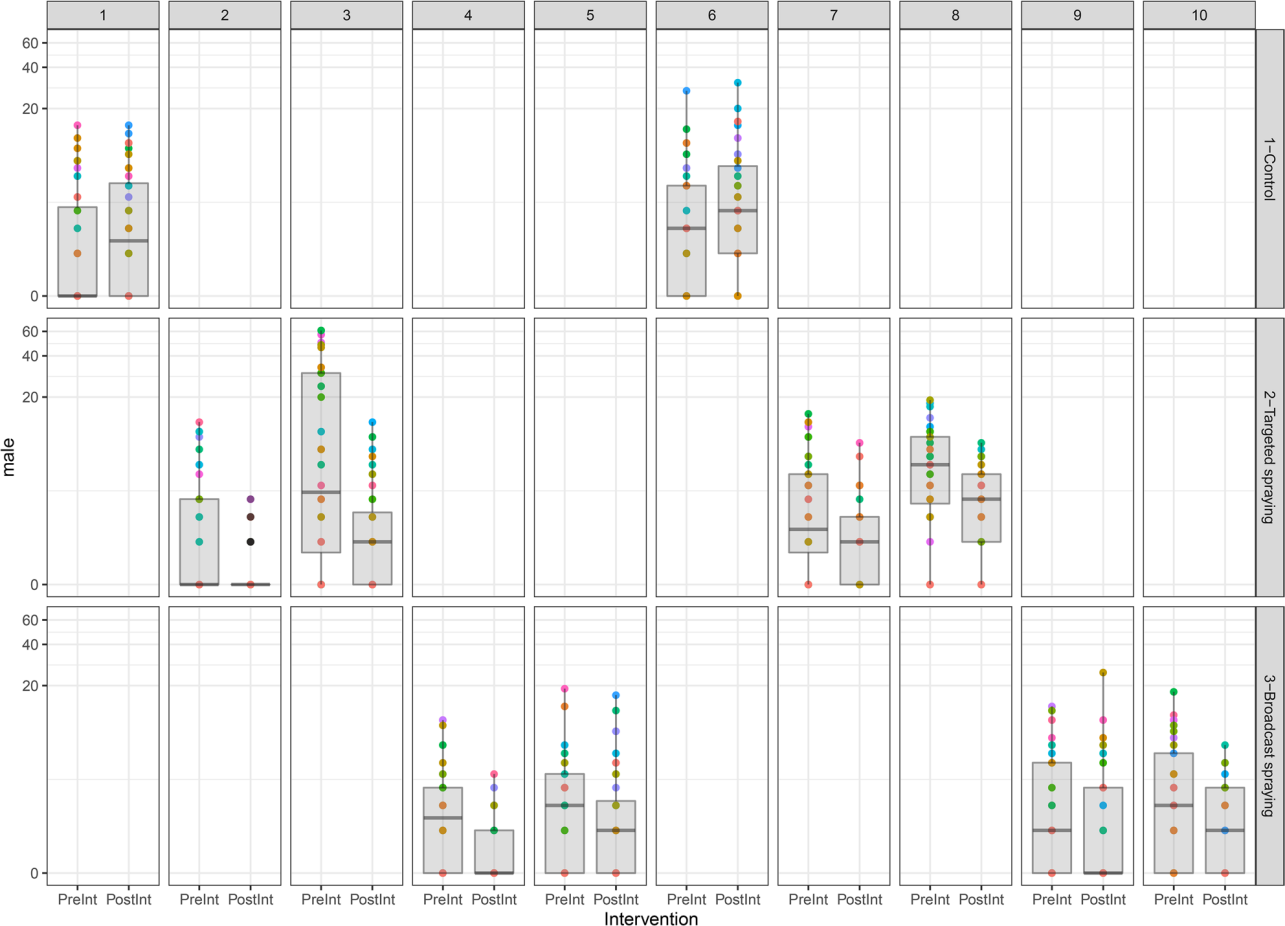
Plot the density of males. In these plots the columns refer to village ID and the rows to intervention type. As each village was only assigned a single intervention each column has only a single filled plot. Each cell shows the density colored by house (points) as well as a boxplot of the distribution of densities over households, both before and after intervention. The y-axis in each plot is given on a log-scale to make the differences in pre- and post-intervention densities more evident. the effect of pre-intervention density had a highly significant negative effect (p-values < 0.001). For change in male density, targeted spraying and broadcast spraying were highly significant with p-value = 0.003 and p-value = 0.001 respectively).

**Figure 9:**
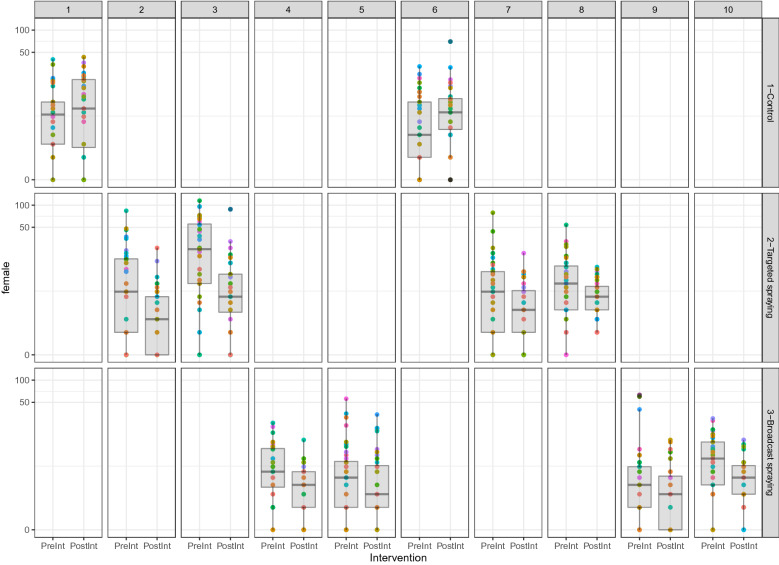
Plot the density of males and females, respectively. In these plots the columns refer to village ID and the rows to intervention type. Each cell shows the density colored by house (points) as well as a boxplot of the distribution of densities over households, both before and after intervention. The y-axis in each plot is given on a log-scale to make the differences in pre- and post-intervention densities more evident. The effect of pre-intervention density had a highly significant negative effect (p-values < 0.001). For the modeled change in female density, coefficients for targeted spraying and broadcast spraying were significant at 0.05 threshold (p-values = 0.003 and p-values = 0.001, respectively).

The effect of intervention type on density of male (Fig. 8) and female (Fig. 9) mosquitoes was analyzed in the generalized estimating equation (GEE) framework. Once again differences between pre- and post-intervention measurements were used as outcome with exchangeable correlation structure. The GEE results for both female and male densities were very similar. In both cases the effect of pre-intervention density had a highly significant negative effect (p-values < 0.001). For the modeled change in female density, coefficients for targeted spraying and broadcast spraying were significant at 0.05 threshold (p-values = 0.003 and p-values = 0.001, respectively). For change in male density, targeted spraying and broadcast spraying were highly significant with p-value = 0.003 and p-value = 0.001 respectively. GLM regressions were run again which resulted in very similar regression coefficients and highly inflated p-values and confidence intervals due to ignoring correlation structure. In the control villages there was an observed mean increase of 45% in mosquito abundance and mean decreases of 68% and 55% in targeted space spray and broadcast space spray villages respectively.

### Impact on the infection rates

**Figure 10:**
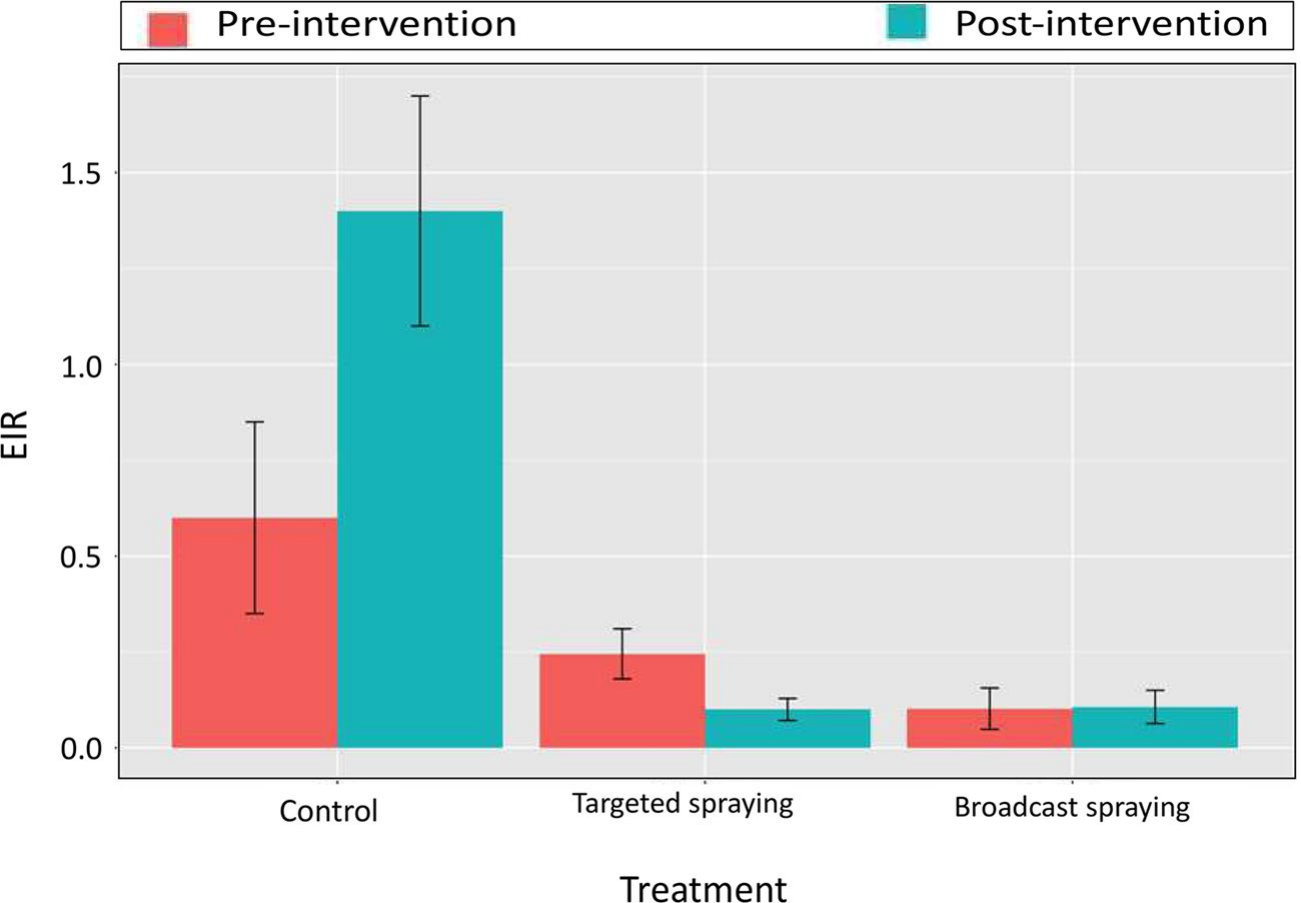
Variation of the Entomological Inoculation Rate (EIR) between intervention periods. A significant reduction was noted in treated villages by targeted spraying (P < 0001).

The Entomological Inoculation Rate (EIR) was higher in the control villages than those treated with either targeted or broadcast spraying. The EIR increased considerably after the intervention period in the control villages, while a trend of reduction was noted in the villages treated with targeted spraying. The values of the EIR were very low in the villages treated by broadcast spraying but no variation was observed between intervention periods (Fig. 10).

## Discussion

The first aim of this study was to demonstrate that swarms of dominant malaria vector species can be readily identified and targeted with effective space spraying, to crash their populations significantly and lower their vectorial capacities, thereby complementing and accelerating efforts towards malaria elimination in the targeted areas. The second aim was to assess the impact of the intervention in order to confirm that the evidence provided in a similar study carried out previously at a small scale^26^, can be generalized as a reliable tool for vector control of malaria.

Prior to assessing the impact of swarm killing, a baseline data collection was conducted from year 1 in the 10 villages, and it was demonstrated that the distribution of potential swarm markers and swarms in villages were clustered across space, making swarm-killing intervention easier^29^. It was also shown that swarm characteristics such as size (averaged 50 mosquitoes per swarm) and height (2 m on average) would respectively facilitate the use of a low quantity of insecticide to kill mosquitoes in swarms and easily reach the whole swarm with appropriate pressure^29^.

The analysis of the amount of insecticide used in the villages of intervention showed a positive correlation between the quantity of insecticide used and the size of the villages where broadcast spraying was used. However, no correlation was found in the villages where the targeted spraying was applied. In fact, the targeted spraying was correlated with the number of swarms. These results confirm that in targeted spraying less insecticides are used than in broadcast spraying with backpack sprayers.

In this study we showed that we can significantly reduce mosquito density by as much as 68% with the targeted swarm spraying. The reduction rate found in this study did not reach the80% reduction in mosquito density recorded during a preliminary study in village VK5 in the western region of Burkina Faso^26^. But this was to be expected because the results of the cluster randomized trial (CRT) power simulations suggested that an intervention resulting in an 80% reduction in mosquito densities could only be achieved with a few numbers of villages as low as two (2 control and 2 intervention villages). On the other hand, if a reduction of less than 40% was expected, up to 10 villages would be required. Given the high impact of swarm destruction observed in the preliminary study, it could be conservatively assumed that an average of 60% reduction in mosquito densities could be achieved. The 68% reduction found in this study with the large number of villages was beyond expectations. These results clearly indicate that targeted swarms spraying offers an unrivalled opportunity to drastically reduce mosquito-density.

In comparison with broadcast spraying as a standard method using backpack sprayers, it can be concluded that both interventions have a highly significant impact, as expected, upon all entomological parameters measured. To see if targeted swarm spraying was significantly more effective than broadcast space spraying, a post-hoc Tukey’s “honest significant differences” test to the fitted GEE models was performed, which is a statistically sound method of post-hoc analysis on fitted models. As the effects of the two interventions were very similar, no significant differences between targeted and broadcast spraying were found for effects on density in male or female, and for insemination or parity rate. However, we found targeted spraying to be significantly more effective than broadcast spraying at reducing number of bites per person per night (NBPN) and entomological insemination rate (EIR) (multiple comparison adjusted p-value = 0.001). The high effect of targeted spraying on the NBPN compared to broadcast spraying could be explained by the massive elimination of virgin females in the swarms. Indeed, after emergence, female mosquitoes are supposed to come in the swarms to get insemination before blood feed seeking. Females are therefore eliminated in the swarm at the same time as the males during the intervention. The elimination of these virgin or inseminated females in the swarms coupled with the elimination of the females looking for a blood meal would have resulted in a drastic decrease of the NBPN in the villages where we have used targeted spraying. On the other hand, the logical consequence of these results was the drastic reduction of mosquito-borne pathogen transmission that we found in the villages where targeted spraying was used.

The results from the 4 villages^29^ treated with the targeted spraying intervention indicated that this strategy could be used to fight against all major malaria vectors in Africa, as they all are characterized by swarm mating behaviour^17–25,35–37^.

## Conclusion

The results revealed a high impact of the targeted swarm spraying intervention on the estimated entomological parameters. The indoor resting mosquito abundance, the age structure of the females and the insemination rates decreased significantly from the pre-intervention to the post-intervention periods. Similarly, the human biting rates and the infection rates (EIR) decreased drastically in the intervention villages. Although targeted swarm spraying and broadcast space spraying are both significantly impactful, lower insecticide quantities were used when targeting swarms directly.

## Data Availability

The raw datasets are available from the corresponding author on reasonable request.

## References

[1] WHO. *World malaria report 2021*. https://www.who.int/publications/i/item/9789240040496 (2021).

[2] Turner SL (2011). Ultra-prolonged activation of CO2-sensing neurons disorients mosquitoes. Nature.

[3] Eisele TP (2012). Estimates of child deaths prevented from malaria prevention scale-up in Africa 2001–2010. Malar. J..

[4] Ulrich JN, Naranjo DP, Alimi TO, Müller GC, Beier JC (2013). How much vector control is needed to achieve malaria elimination?. Trends Parasitol..

[5] Reddy MR (2011). Outdoor host seeking behaviour of Anopheles gambiae mosquitoes following initiation of malaria vector control on Bioko Island Equatorial Guinea. Malar. J..

[6] Russell TL (2011). Increased proportions of outdoor feeding among residual malaria vector populations following increased use of insecticide-treated nets in rural Tanzania. Malar. J..

[7] Moiroux N (2012). Changes in anopheles funestus biting behavior following universal coverage of long-lasting insecticidal nets in benin. J. Infect. Dis..

[8] Okumu FO (2013). Comparative field evaluation of combinations of long-lasting insecticide treated nets and indoor residual spraying, relative to either method alone, for malaria prevention in an area where the main vector is Anopheles arabiensis. Parasit. Vectors.

[9] The malERA Consultative Group on Vector Control (2011). A research agenda for malaria eradication: Vector control. PLoS Med..

[10] Alonso PL (2011). A research agenda to underpin malaria eradication. PLoS Med..

[11] Clements, A. N. *The Biology of Mosquitoes, Volume 3 Transmission of Viruses and Interactions with Bacteria*. (CABI, 1992).

[12] Clements A (1999). The Biology of Mosquitoes. Sensory Reception and Behavior.

[13] Ferguson HM (2010). Ecology: A prerequisite for malaria elimination and eradication. PLoS Med..

[14] Ferguson HM, John B, Nghabi K, Knols BGJ (2005). Redressing the sex imbalance in knowledge of vector biology. Trends Ecol. Evol..

[15] Godfray HCJ (2013). Mosquito ecology and control of malaria. J. Anim. Ecol..

[16] Diabate A, Tripet F (2015). Targeting male mosquito mating behaviour for malaria control. Parasit. Vectors.

[17] Diabaté A (2009). Spatial swarm segregation and reproductive isolation between the molecular forms of Anopheles gambiae. Proc. R. Soc. B Biol. Sci..

[18] Diabaté A (2006). Mixed swarms of the molecular M and S forms of Anopheles gambiae (Diptera: Culicidae) in sympatric area from Burkina Faso. J. Med. Entomol..

[19] Diabaté A (2011). Spatial distribution and male mating success of Anopheles gambiae swarms. BMC Evol. Biol..

[20] Assogba BS (2014). Characterization of swarming and mating behaviour between Anopheles coluzzii and Anopheles melas in a sympatry area of Benin. Acta Trop..

[21] Charlwood JD, Jones MDR (1979). Mating behaviour in the mosquito, Anopheles gambiae s.1.save: I. Close range and contact behaviour. Physiol. Entomol..

[22] Charlwood JD, Jones MDR (1980). Mating in the mosquito, Anopheles gambiae s.l.. Physiol. Entomol..

[23] Charlwood JD (2002). The swarming and mating behaviour of Anopheles gambiae s.s. (Diptera: Culicidae) from São Tomé Island. J. Vec. Ecol..

[24] Charlwood JD, Thompson R, Madsen H (2003). Observations on the swarming and mating behaviour of Anopheles funestus from southern Mozambique. Malar. J..

[25] Marchand RP (1984). Field observations on swarming and mating in anopheles gambiae mosquitoes in tanzania1. Netherlands J. Zool..

[26] Sawadogo SP (2017). Targeting male mosquito swarms to control malaria vector density. PLoS ONE.

[27] Dabire KR (2013). Assortative mating in mixed swarms of the mosquito anopheles gambiae s.s. m and s molecular forms, in burkina faso, west africa. Med. Vet. Entomol..

[28] Lees RS (2014). Review: Improving our knowledge of male mosquito biology in relation to genetic control programmes. Acta Trop..

[29] Niang A (2021). Entomological baseline data collection and power analyses in preparation of a mosquito swarm-killing intervention in south-western Burkina Faso. Malar. J..

[30] Wu L (2009). Mixed Effects Models for Complex Data.

[31] Alain FZ, Elena NI, Neil JW, Anatoly AS, Smith M (2009). Mixed Effects Models and Extensions in Ecology with R.

[32] Team, R. C. A Language and Environment for Statistical Computing. (2013).

[33] Halekoh U, Højsgaard S, Yan J (2006). The R package geepack for generalized estimating equations. J. Stat. Softw..

[34] Wickham H (2019). Welcome to the Tidyverse. J. Open Source Softw..

[35] Sawadogo SP (2013). Differences in timing of mating swarms in sympatric populations of Anopheles coluzzii and Anopheles gambiae ss (formerly An gambiae M and S molecular forms) in Burkina Faso, West Africa. Parasit. Vect..

[36] Sawadogo PS (2014). Swarming behaviour in natural populations of Anopheles gambiae and An. coluzzii: Review of 4 years survey in rural areas of sympatry, Burkina Faso (West Africa). Acta Trop..

[37] Downes JA (1969). The swarming and mating flight of diptera. Annu. Rev. Entomol..

